# INTERRUPTED TALK: Exploring the barriers encountered by psychiatry trainees with utilising a CBT-based framework in patient clinical encounters in an NHS Trust – A qualitative study

**DOI:** 10.1192/j.eurpsy.2025.918

**Published:** 2025-08-26

**Authors:** A. Obiekezie, F. Dewsnap, J. Anderson

**Affiliations:** 1 SABP, Surrey; 2 Brighton and Sussex Medical School, Brighton, United Kingdom

## Abstract

**Introduction:**

The UK Royal College of Psychiatrists and the General Medical Council both recognise the value of and commend the use of Cognitive Behavioural Therapy (CBT) in understanding and treating Psychiatric disorders. It is now mandatory to incorporate in CBT in psychiatry training. Previous research and the authors’ own experiences as a trainee have shown that despite being trained in CBT, there continues to be limited use of CBT in routine clinical practice by psychiatry trainees. This study was conducted at a UK NHS Trust as an educational service evaluation.

**Objectives:**

Despite trainees receiving extensive CBT training and completing a 12-session CBT case early in their training, many do not use this skill. The objective of the study was to explore any barriers psychiatry trainees encountered in utilising CBT in routine clinical practise.

**Methods:**

A qualitative, ethnographic approach using focus group discussions was used. Three Focus groups were conducted. These were audio-recorded and transcribed verbatim, then analysed using a General Thematic Analysis. A coding framework was used to organise emergent themes into five broad categories and are shown in the results section.

**Results:**

**Table 1:** Barriers to CBT use

**Training Factors**	**Patient Factors**	**Psychiatrist/Clinician Factors**	**Systemic Factors**	**Other Factors**
Not taught in medical schools Insufficient training Training too abstract Time-lag between training and practice Lack of access to information on further training Trainers’ emphasis No familiarity with integrating into assessments	Patient Willingness Suitability Patient preference for medication Patient mindset about psychiatrist’s role Not considered tangible by patients Poor rapport with medics in in-patient settings Severity of illness	Few advocates Subspecialisation Lack of confidence Unavailability of senior clinicians Culture of clinicians Lack of autonomy Lack of practise Not part of Doctor’s role	Time Type of care setting (Community vs Inpatient) Pressure on services. No senior modelling Lack of clarity of concept Focus on other areas at assessment. Lack of supervision No therapist continuity or formalised follow-up	CBT is too structured

**Conclusions:**

Psychiatry trainees were eager to use their CBT skills more often yet find barriers hindering their aspirations. In a specialty where talking with patients can sometimes be as effective as offering them medications, having a deeper understanding of the hindrances trainees encounter with regularly deploying this skill is crucial.

**Disclosure of Interest:**

None Declared

